# The Role of Exosomes in Human Carcinogenesis and Cancer Therapy—Recent Findings from Molecular and Clinical Research

**DOI:** 10.3390/cells12030356

**Published:** 2023-01-18

**Authors:** Katarzyna Stefańska, Małgorzata Józkowiak, Ana Angelova Volponi, Jamil Awad Shibli, Afsaneh Golkar-Narenji, Paweł Antosik, Dorota Bukowska, Hanna Piotrowska-Kempisty, Paul Mozdziak, Piotr Dzięgiel, Marzenna Podhorska-Okołów, Maciej Zabel, Marta Dyszkiewicz-Konwińska, Bartosz Kempisty

**Affiliations:** 1 Department of Histology and Embryology, Poznan University of Medical Sciences, 60-781 Poznan, Poland; 2 Cellivia 3 S.A., 61-623 Poznan, Poland; 3 Department of Toxicology, Poznan University of Medical Sciences, 60-631 Poznan, Poland; 4 Centre for Craniofacial and Regenerative Biology, Dental Institute, King’s College London, London WC2R 2LS, UK; 5 Department of Periodontology and Oral Implantology, University of Guarulhos, Guarulhos 07030-010, Brazil; 6 Prestage Department of Poultry Sciences, North Carolina State University, Raleigh, NC 27695, USA; 7 Department of Veterinary Surgery, Institute of Veterinary Medicine, Nicolaus Copernicus University in Torun, 87-100 Torun, Poland; 8 Department of Diagnostics and Clinical Sciences, Institute of Veterinary Medicine, Nicolaus Copernicus University in Torun, 87-100 Torun, Poland; 9 Department of Basic and Preclinical Sciences, Institute of Veterinary Medicine, Nicolaus Copernicus University in Torun, 87-100 Torun, Poland; 10 Division of Histology and Embryology, Department of Human Morphology and Embryology, Wroclaw Medical University, 50-368 Wroclaw, Poland; 11 Division of Ultrastructural Research, Department of Human Morphology and Embryology, Wroclaw Medical University, 50-368 Wroclaw, Poland; 12 Division of Anatomy and Histology, University of Zielona Góra, 65-046 Zielona Góra, Poland; 13 Department of Biomaterials and Experimental Dentistry, Poznan University of Medical Sciences, 60-812 Poznan, Poland; 14 Division of Anatomy, Department of Human Morphology and Embryology, Wroclaw Medical University, 50-368 Wroclaw, Poland; 15 Department of Obstetrics and Gynecology, University Hospital and Masaryk University, 601 77 Brno, Czech Republic

**Keywords:** exosomes, cancer, drug resistance, cancer therapies

## Abstract

Exosomes are biological nanoscale spherical lipid bilayer vesicles, 40–160 nm in diameter, produced by most mammalian cells in both physiological and pathological conditions. Exosomes are formed via the endosomal sorting complex required for transport (ESCRT). The primary function of exosomes is mediating cell-to-cell communication. In terms of cancer, exosomes play important roles as mediators of intercellular communication, leading to tumor progression. Moreover, they can serve as biomarkers for cancer detection and progression. Therefore, their utilization in cancer therapies has been suggested, either as drug delivery carriers or as a diagnostic tool. However, exosomes were also reported to be involved in cancer drug resistance via transferring information of drug resistance to sensitive cells. It is important to consider the current knowledge regarding the role of exosomes in cancer, drug resistance, cancer therapies, and their clinical application in cancer therapies.

## 1. The Structure and Sources of Exosomes

Exosomes are biological nanoscale spherical lipid bilayer vesicles, 40–160 nm in diameter, produced by most mammalian cells in both physiological and pathological conditions [1,2]. Exosomes were found in various body fluids, such as saliva [3], semen [4], plasma [5], urine [6], amniotic fluid [7], bile [8], and others. This review aims to summarize the current knowledge regarding the role of exosomes in cancer, drug resistance, cancer therapies, and their clinical application. Therefore, we performed an electronic search in the PubMed database using search terms such as ‘exosomes’, ‘exosome biogenesis’, ‘exosomes function’, ‘exosomes cancer’, ‘human carcinogenesis’, ‘exosomes cancer therapies’, and ‘exosomes drug resistance’. We also searched the ClinicalTrials.gov database to find relevant clinical trials.

Exosomes represent a subset of extracellular vesicles (EVs) along with ectosomes–vesicles which are generated by outward budding from the plasma membrane, followed by the membrane being pinched off and released to the extracellular space. Ectosomes comprise microvesicles (MVs), which are 100–1000 nm in size and contain cytoplasmic cargo, and apoptotic bodies, which are 50 nm–2 µm in size and are released by dying cells [9].

**Figure 1:**
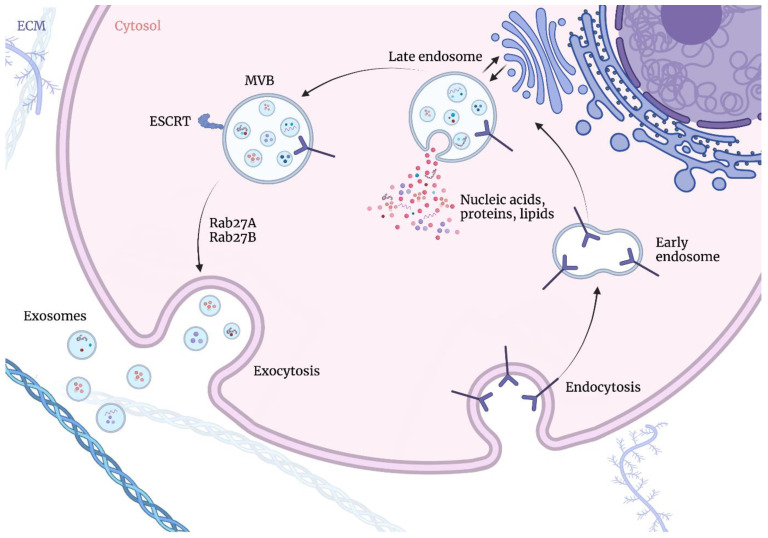
Exosome biogenesis occurs via endosomal route beginning with the invagination of the plasma membrane and the formation of early-sorting endosomes (ESEs) containing cell surface proteins and soluble extracellular proteins, with the participation of the endoplasmic reticulum, the trans-Golgi network, and the mitochondria. The endoplasmic reticulum and the trans-Golgi network may also contribute to ESEs maturing into late-sorting endosomes (LSEs). The second invagination of LSEs gives rise to intraluminal vesicles (ILVs) with cytoplasmic constituents and proteins. Subsequently, LSEs give rise to intercellular multivesicular bodies (MVBs) containing ILVs, through an inward invagination of the endosomal-limiting membrane. MVBs may then be transported to the plasma membrane via the cytoskeletal and microtubule network and dock on the luminal side of the plasma membrane, which is followed by exocytosis. Figures were created with BioRender.com (accessed on 1 November 2022).

On the contrary, exosomes are formed via the endosomal route [10], beginning with the invagination of the plasma membrane and the formation of early-sorting endosomes (ESEs) containing cell surface proteins and soluble extracellular proteins, with participation of the endoplasmic reticulum, the trans-Golgi network, and the mitochondria. The endoplasmic reticulum and the trans-Golgi network may also contribute to the ESEs maturing into late-sorting endosomes (LSEs). The second invagination of the LSEs gives rise to intraluminal vesicles (ILVs) with cytoplasmic constituents and proteins. Subsequently, LSEs give rise to intercellular multivesicular bodies (MVBs) containing ILVs, through an inward invagination of the endosomal limiting membrane. MVBs may then be transported to the plasma membrane via the cytoskeletal and microtubule network and dock on the luminal side of the plasma membrane, which is followed by exocytosis [2]. Therefore, exosomes correspond to the ILVs of MVBs. The biogenesis of exosomes is presented in Figure 1.

The formation of MVBs and ILVs may occur via an ubiquitin-dependent or ubiquitin-independent pathway. The ubiquitin-dependent mechanism is considered the most important pathway of exosome formation, involving the endosomal sorting complex required for transport (ESCRT), which sorts ubiquitinated proteins into ILVs [11]. ESCRT comprises four complexes: ESCRT-0, ESCRT-I, ESCRT-II, and ESCRT-III with their associated proteins (vacuolar protein sorting-associated protein 4 (VPS4), vacuolar sorting-associated protein 1 (VTA1), and ALIX). ESCRT-0 is activated by phosphatidylinositol 3-phosphate PI(3)P and comprises the hepatocyte growth-factor-regulated tyrosine kinase substrate (HRS), which is able to recognize monoubiquitylated cargo proteins and recruits ESCRT-1 via the interaction with its subunit (tumor susceptibility gene 101 (TSG101)) [12]. Subsequently, ESCRT-I interacts with ESCRT-II or ALIX (which are responsible for membrane deformation into buds [12]) in order to recruit ESCRT-III. ESCRT-II was also demonstrated to initiate the assembly of the ESCRT-III complex, which is involved in vesicle scission and ESCRT recycling via interaction with the AAA-ATPase VPS4 [12,13]. Besides the aforementioned proteins, syntenin-1, syndecan-1, the Ras-related protein GTPase Rab, and the soluble N-ethylmaleimide-sensitive fusion attachment protein receptor (SNARE) complexes were demonstrated to participate in exosome biogenesis as well [14].

Some studies indicate that exosome formation may occur in an ESCRT-independent manner. Stuffers et al. [15] depleted HEp-2 cells of key subunits of four ESCRTs via siRNAs, which resulted in the morphology of endocytic structures becoming altered. However, the formation of ILVs and MVBs still occurred, suggesting that another mechanism is involved with other than ESCRTs. Trajkovic et al. [16] revealed an alternative pathway for sorting cargo into MVBs and participating in exosome formation. The authors interfered with the function of ESCRT machinery via RNAi or the depletion of HRS, TSG101, ALIX, and VPS4 in oligodendroglial cells that secrete exosomes containing proteolipid proteins (PLPs), which did not impair exosome secretion. Since purified exosomes were enriched in ceramide and given that the inhibition of neutral sphingomyelinases reduced the release of exosomes, it seems that the formation of this class of exosomes requires the sphingolipid ceramide [16]. Chairoungdua et al. [17] demonstrated that transmembrane proteins enriched in exosomes–tetraspanins, such as CD82 and CD9, regulate the biogenesis of exosomes. Both these proteins were shown to reduce the cellular pool of β-catenin by enhancing its exosome-mediated discharge from the cells [17]. Nazarenko et al. [18] employed rat pancreatic adenocarcinoma cells to suggest that Tspan8 affects the protein composition of exosomes. A tetraspanin, CD63, was suggested to play an important role in exosome biogenesis, as indicated by Hurwitz et al. [19]. The knockout of CD63 via CRISPR/Cas9 resulted in a reduction in the secretion of EVs. CD63 was also demonstrated to play an important role in the exosomal packaging of latent membrane protein 1 (LMP1) [20]. Perez-Hernandez et al. [21] revealed that the ligands of the intracellular tetraspanin-enriched microdomain (TEM) and CD81 may play a crucial role in incorporating proteins into the exosome structure. The small integral membrane protein of the lysosome–late endosome (SIMPLE) was demonstrated to reside within the ILVs of MVBs and exosomes. As indicated by Zhu et al. [22], the SIMPLE regulates the production of exosomes by modulating the formation of MVBs. Therefore, there are several mechanisms of exosome biogenesis, either with or without the participation of the ESCRT complex.

**Figure 2:**
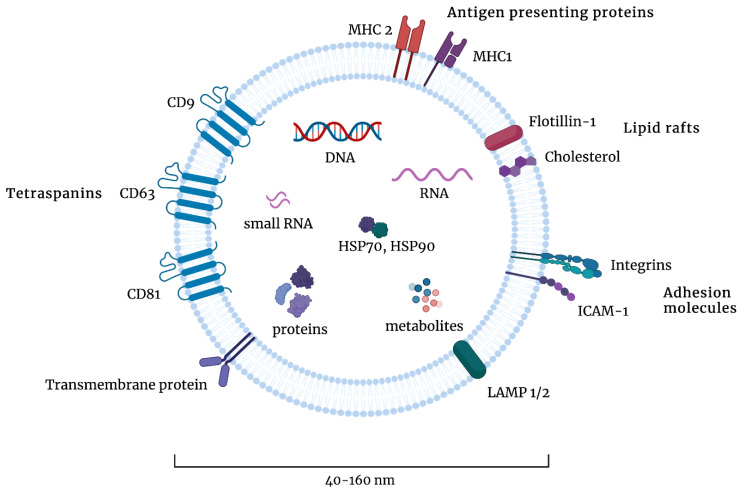
The composition of exosomes. Exosomes are 40–160 nm in diameter and contain various types of proteins, such as tetraspanins, transmembrane proteins, adhesion molecules, lipid rafts, or antigen-presenting proteins. They contain DNA, RNA, small RNA, heat shock proteins (HSPs), and other metabolites. Figures were created with BioRender.com (accessed on 1 November 2022).

Exosomal content is highly dependent on their cellular origin; however, most exosomes contain proteins involved in MVB biogenesis (such as ALIX, TSG101, VPS, and clathrin), tetraspanins (such as CD9, CD63, CD81, and CD82), heat shock proteins (such as HSP70 or HSP90), and adhesion and signal transduction proteins (such as integrins, flotillin, and syntenin-1) [23,24]. The composition of exosomes is presented in Figure 2. The proteins that most frequently are present in exosomes are deposited in online databases, such as ExoCarta, EVPedia, or Vesiclepedia [25,26,27].

## 2. The Cellular Function of Exosomes

It seems that the most important function of exosomes is mediating cell-to-cell communication. Initially, exosomes were assumed to be involved in cellular waste removal [28,29]. Exosomes contain various metabolites, proteins, lipids, and nucleic acids, but their composition may be altered via cellular conditions and is dependent on the cell type [30]. Exosomes may affect acceptor cells, either by interacting with extracellular receptors or by being uptaken [31].

During immunomodulatory or apoptotic signaling, exosomal surface ligands bind directly with the surface receptor on the recipient cell, activating a subsequent signaling cascade [31]. For example, exosomes secreted by dendritic cells possess MHC peptide complexes enabling T-cell activation [32]. They also express surface ligands such as the tumor necrosis factor (TNF), the Fas ligand (FasL), and the TNF-related apoptosis-inducing ligand (TRAIL), meaning that they can bind to TNF receptors on tumor cells, triggering caspase activation and tumor apoptosis. Moreover, dendritic-cell-derived exosomes activate natural killer (NK) cells by interacting with its TNF receptor, leading to interferon γ (IFNγ) secretion [33]. Therefore, exosomes stimulate innate immune responses.

In other cases, exosomes may fuse with the plasma membrane of target cells, resulting in the delivery of their cargo to the cytoplasm. The internalization of exosomes may occur via a specific receptor-dependent pathway or non-specific micro- or macropinocytosis. Integrins, lectins, proteoglycans and T-cell immunoglobin, mucin-domain-containing protein 4 (Tim4), as well as synctin-1 and syncytin-2 are likely to participate in exosome uptake [14,34]. Endocytosis occurring during exosome internalization may be dependent or independent on clathrin-mediated endocytosis, which involves the assembly of clathrin-coated pits. Subsequently, clathrin-coated vesicles become uncoated and fuse with endosomes [35]. The mechanisms of clathrin-independent exosome endocytosis include lipid-raft-mediated endocytosis, caveolin-mediated endocytosis, and phagocytosis. Importantly, these pathways may coexist during exosome internalization and the released cargo bypasses direct lysosomal degradation via multiple routes [31].

In terms of cancer, exosomes play important roles as mediators of intercellular communication, leading to tumor progression [36]. Furthermore, they can serve as biomarkers for cancer detection and progression [37]. Cancer cells were shown to secrete exosomes that influence cancer progression via the formation of tumor-promoting stroma and the differentiation of fibroblasts towards tumor-promoting stromal myofibroblasts [38]. Such differentiation was dependent on triggering α-smooth muscle actin expression and TGF-β signaling [39]. Cancer-associated fibroblasts (CAFs) play an important role in cancer’s aggressiveness, but they also secrete exosomes that participate in the invasiveness of cancer cells. Exosomes derived from CAFs stimulated protrusive activity and the motility of breast cancer cells through Wnt–planar cell polarity (Wnt-PCP) signaling [40]. Cho et al. [41] obtained exosomes from ovarian cancer cell lines—SK-OV-3 and OVCAR-3—and introduced them to adipose-tissue-derived mesenchymal stem cells (ADSCs), resulting in myofibroblastic functionality and ADSC phenotypes [41]. Therefore, exosomes secreted by cancer cells or CAFs directly influence their surrounding stroma and other cells, changing their functionality and promoting cancer progression.

Cancer-cell-derived exosomes also exert immunoregulatory functions, leading to the immune suppression or avoidance of immune responses. Clayton et al. [42] demonstrated that the proliferation of peripheral blood lymphocytes in response to IL-2 was inhibited due to cancer-derived exosomes and the activity of NK cells was decreased. Yu et al. [43] revealed that TS/A murine mammary tumor cells secreted exosomes that block the differentiation of tumor myeloid precursor cells into dendritic cells due to the induction of IL-6. Exosomes derived from human ovarian cancer specimens suppressed the expression of signaling components of T cell activation, such as JAK3 and CD3-*ζ*; moreover, they induced T cell apoptosis, as indicated by Taylor et al. [44]. In vitro studies have suggested that cancer-cell-derived exosomes influence gene expression in T cells, inducing the upregulation of inhibitory genes in CD4+ T cells, leading to a loss of CD69 and functional decline, although exosomes are not internalized by T cells [45]. Colorectal cancer cells were shown to secrete exosomes containing miR-145, which were incorporated by macrophages, leading to their polarization towards the M2 phenotype [46].

Exosomes secreted by cancer cells may play an important role in angiogenesis, which is crucial for cancer progression. Melanoma cells were demonstrated to release exosomes containing factors regulating angiogenesis, such as the vascular endothelial growth factor (VEGF), IL-6, and matrix metalloproteinase 2 (MMP2), due to WNT5A signaling. Moreover, the depletion of WNT5A resulted in decreased endothelial cell branching [47]. Umezu et al. [48] revealed that leukemia cell (K562)-derived exosomes contain the miR-17-92 cluster, which can be transported to endothelial cells (HUVECs—human umbilical vein endothelial cells), leading to the decreased expression of integrin α5. Therefore, exosomes are able to participate in leukemia–endothelial cell communication due to their miRNA cargo. Similarly, colorectal cancer cells promote the proliferation of endothelial cells via exosomes and their mRNA content [49]. Metastatic cancer cells secrete exosomes containing miR-105, which targets the tight junction protein zonula occludens-1 (ZO-1) leading to vascular permeability [50]. Zhang et al. [51] revealed that renal cancer-cell-derived exosomes promote angiogenesis in HUVECs via the upregulation of VEGF expression. These findings suggest that exosomes secreted by cancer cells are involved in both angiogenesis and metastasis during disease progression.

**Figure 3:**
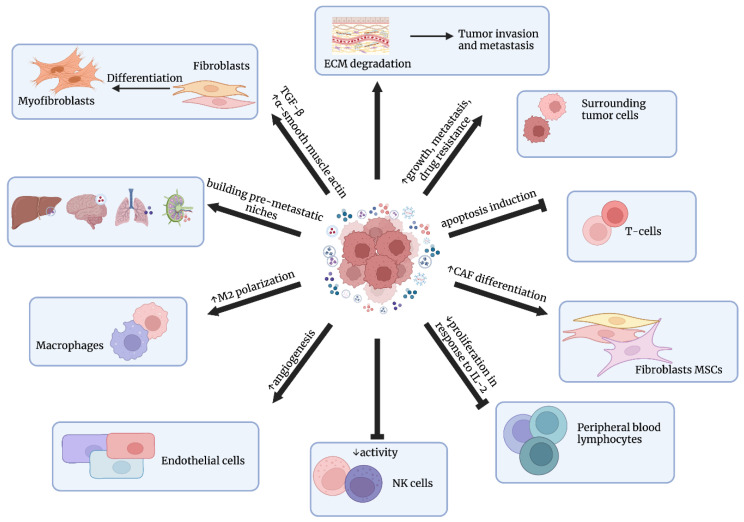
The influence of exosomes on cancer progression. Exosomes participate in building pre-metastatic niches, angiogenesis, ECM degradation, growth, metastasis, and drug resistance; they also regulate immune cells’ function. Figures were created with BioRender.com (accessed on 1 November 2022).

Cancer-cell-derived exosomes also influence extracellular matrix (ECM) and play a crucial role in the movement of cancer cells via promoting adhesion assembly. The fibronectin contained in these exosomes seems to be particularly important for cell migration [52]. Mu et al. [53] revealed that tumor-derived exosomes are able to bind to individual components of the ECM, e.g., hyaluronic acid or laminin. Moreover, these exosomes are rich in proteases, which can degrade collagens, laminins, or fibronectin, leading to premetastatic niche preparation [53]. Metastatic capability can be transferred between metastatic and nonmetastatic cancer cells via exosomes, as indicated by Le et al. [54]. Extracellular vesicles containing miR-200, secreted by metastatic breast cancer cell lines, were shown to alter gene expressions and promote the mesenchymal-to-epithelial transition (MET) in nonmetastatic cells [54]. Another example is pancreatic ductal adenocarcinoma, as exosomes were demonstrated to induce liver premetastatic niche formation in naïve mice. TGF-β secretion and fibronectin upregulation in recipient hepatic cells, creating fibrotic microenvironment, while the macrophage migration inhibitory factor (MIF) contained within exosomes counteracted against bone-marrow-derived macrophages leading to metastasis [55]. Exosomal integrins seem to be particularly important in determining organ-specific metastasis, since integrins α_6_β_4_ and α_6_β_1_ were associated with lung metastasis, whereas integrin α_V_β_4_ was linked with liver metastasis [56]. The influence of exosomes on cancer progression is presented in Figure 3.

## 3. Factors Influencing Human Carcinogenesis

Cancer has been present in human species for thousands of years, suggesting that this is not an exclusively civilization-based disease [57]. Carcinogenesis requires the complex interplay of both exogenous and endogenous factors influencing many critical signaling pathways, resulting in genetic instability. However, one of the earliest hypotheses concerning the etiology of carcinogenesis was chemical carcinogenesis. Chemicals called carcinogens, such as polycyclic aromatic hydrocarbons, were found to be able to bind to cellular DNA. Their electrophilic intermediates were able to form covalent bonds with nucleophilic regions on nucleotides, which could lead to encoded gene alterations and a transformation into cancer cells. The *TP53* gene encoding the tumor suppressor protein or the retinoblastoma (*RB*) is particularly sensitive to such mutations [58]. The metabolic activation of carcinogens was shown to occur via hepatic enzymes, such as the monooxygenase cytochrome P450 or CYP. Subsequently, it was established that endogenous molecular pathways may also cause mutations in specific genes via reactive oxygen species (ROS) production, leading to cell cycle destabilization [59]. Moreover, the cellular soluble receptors (such as the estrogen receptor—ER) may be modulated via carcinogens due to their structural similarities with natural chemicals, which may lead to normal signaling pathway alterations. Increased proliferation or decreased apoptosis, occurring due to abnormalities in the signaling pathways, may eventually lead to cancer cells expanding into tumors. Signaling pathways mediated by RAS, ERK, PDPK1, and TP53 are often altered in various types of cancer, as they are involved in processes such as apoptosis, cell proliferation, and migration or cell differentiation [58].

Besides chemical carcinogens, viruses may also play a role in the pathogenesis of some human cancers. Several DNA viruses are associated with specific types of cancer. The hepatitis B virus (HBV) is a risk factor for hepatocellular carcinoma; some types of human papillomavirus (HPV) are associated with ureteral, penile, and upper aerodigestive tract carcinomas; Epstein–Barr virus (EBV) is a risk factor for Burkitt lymphoma, Hodgkin and non-Hodgkin lymphoma; and Kaposi sarcoma-associated herpesvirus (KSHV) is associated with Kaposi sarcoma [60]. Infection with these viruses may cause genetic instability; for example, the human papillomavirus type 16 E7 oncoprotein can induce abnormal centrosome duplication through a mechanism dependent on the inactivation of retinoblastoma protein family members [61]. The HPV E6 oncoprotein enhances the ubiquitin-dependent proteolysis of the O^6^-methylguanine-DNA methyltransferase (MGMT) enzyme, which is involved in DNA repair [62].

Viruses may also lead to cell immortalization or intense proliferation, e.g., EBV can cause the immortalization of B-lymphoblastoid cell lines due to an increase in telomerase activity and a change in the karyotype from diploids to aneuploids [63]. Such events may drive the acquisition of a malignant phenotype. Similarly, viruses may target important cellular regulatory proteins, such as p53. For example, E6 proteins from HPV-16 or HPV-18 cause the proteasomal degradation of p53 [60].

Reactive oxygen species (ROS) are also factors known to induce mutations in purines, pyrimidines, or oxidate proteins, leading to genomic instability and eventually cancer. In normal conditions, ROS can be attenuated with antioxidant enzymes, such as catalase or superoxide dismutase (SOD). ROS production occurs during the exposition to many carcinogens, including cigarette smoke, ethanol consumption, benzene, or polychlorinated biphenyls, and it is associated with tumor-promoting effects during the early stages of carcinogenesis. Besides exposition to carcinogens, obesity is also a factor causing increased ROS production, due to persistent inflammation. Infectious agents such as HBV, EBV, or HPV were also demonstrated to induce ROS production [64]. For example, HPV oncogenic proteins were shown to increase NADPH oxidase (NOX) and decrease antioxidant enzymes [65]. ROS may also act as second messengers or modify proteins that participate in signaling pathways associated with carcinogenesis, such as the nuclear factor kappa-light-chain-enhancer of activated B cells (NF-κB), mitogen-activated protein kinases (MAPKs), hypoxia inducible factor 1 alpha (HIF-1α), phosphatidylinositol 3-kinase (PI3K), nuclear factor-erythroid factor 2-related factor 2 (NRF2), and p53 pathways [64].

The development of sporadic cancers can be explained by two main theories: the somatic mutation theory (SMT) and the tissue organization field theory (TOFT). The SMT assumes that cancer is derived from a single somatic cell that accumulates multiple mutations, whereas the source of cancer is a loss of tissue organization according to the TOFT. However, each theory has its flaws. Therefore, there are hybrid theories which aim to meld the SMT with the concept which indicates that cancer also occurs due to abnormal tissue organization [66].

The tumor microenvironment (TME) is a structural and dynamic network of cellular and non-cellular interactions between cancer cells and the surrounding non-malignant matrix (TME) [67]. The TME is closely related to carcinogenesis, progression, metastasis and drug resistance due to dynamic interactions with cancer cells. It also serves as sustained niche for cancer cells to proliferate and metastasize [67]. The TME consists of multiple cell types (e.g., fibroblasts, immune cells, endothelial cells, pericytes, and MSCs), which are all embedded [67]. The TME consists of multiple cell types (e.g., fibroblasts, immune cells, endothelial cells, pericytes, and MSCs), which are all embedded in the ECM [68]. Recent developments suggest that exosomes are also a part of the TME and play an important role in effective cellular communication between cancer cells and surrounding cells [69,70]. Depending on their cargo, cancer-derived exosomes promote or suppress tumor cell progression, induce chronic inflammation, or lead to immune evasion [71]. In addition, exosomes secreted by cancer cells have been associated with the promotion of angiogenesis [72].

## 4. The Role of Exosomes in Cancer Therapies

Since exosomes were implicated in cell-to-cell communication, their utilization in cancer therapies has been suggested. There are two main applications of exosomes: disease diagnosis and exosome-mediated drug delivery. Exosomes derived from cancer cells are rich in biomarkers that differ from those released by normal cells. Their cargo reflects the content of the cell that they are derived from. Therefore, liquid biopsy followed by exosomal analysis may be useful in monitoring disease progression, differentiating the cancer type, as well as predicting the patient’s survival or response to anti-cancer treatments [73]. In addition, exosomes may be used as biomarkers of non-cancerous conditions, such as gestational diabetes; therefore, their diagnostic application is not limited to cancer [74].

To date, several techniques of exosome isolation have been developed. It seems that in the case of biofluids, ultracentrifugation techniques are the most useful and are considered the gold standard. Ultracentrifugation or density gradient centrifugation were successfully utilized to isolate exosomes from serum, urine, cerebrospinal fluid, breast milk, amniotic fluid, or saliva [75]. However, the ultracentrifugation protocol should be optimized for a specific sample type in order to obtain a high yield and the purity of exosomes. It is also important to consider that ultracentrifugation is time-consuming and that exosomes might be damaged due to centrifugation at high speeds [76]. Therefore, the development of scalable and reproducible methods for exosome isolation still remains a challenge. Besides ultracentrifugation, size-based techniques are also gaining popularity among researchers, providing a higher particle yield and isolation efficiency. Importantly, ultrafiltration or size exclusion chromatography (SEC) may be utilized in the case of exosome isolation from serum and plasma [75]. Exosomes might also be isolated via immunoaffinity techniques, e.g., due to employing specific antibody-coated magnetic beads, which was proven to be more efficient than ultracentrifugation. However, this technique is costly and not suitable on a large scale. Finally, the precipitation techniques utilizing polymers such as polyethylene glycol seem promising for clinical utilization due to their simplicity and no risk of damaging the exosomes. However, such precipitation might result in contamination from plasma proteins [75]. Every aforementioned method has its advantages and disadvantages; therefore, it is important to optimize the isolation protocols and adjust them for a specific sample and intended use.

Although there is still a need to perform further studies in order to develop cancer biomarkers based on cancer-derived exosomes, the research in recent years has provided some promising results. The utilization of exosomes as cancer diagnostic biomarkers seems particularly beneficial, since they might be obtained in a minimally invasive manner, especially from body fluids such as saliva or urine. The collection and enrichment of exosomes may occur due to their surface proteins. The concentration of exosomes is higher than circulating tumor cells, which are also used in liquid biopsies; therefore, their acquisition does not require a large amount of specific body fluid. Exosomes also possess higher stability in the bloodstream than circulating tumor DNA often used for liquid biopsies [77]. Such exosomal biomarkers may not only be used for the distinction of cancer and non-cancer, but also to monitor the disease during therapy or to predict the tumor’s response to treatment. Moreover, the exosomal cargo is highly specific and depends on the cancer type or stage. For example, McKiernan et al. [78] developed a urine gene expression assay to predict high-grade prostate cancer, discriminating high grades (≥Gleason score (GS7) from low grades (GS6) and benign diseases. As a result, unnecessary biopsies could be avoided, as well as the overdiagnosis and overtreatment of prostate cancer [78]. Therefore, the development of diagnostic biomarkers based on exosomes would certainly improve patients’ care and the accuracy of diagnosis. Exosomal cargo that might be utilized as biomarkers in cancer comprises nucleic acids (miRNAs, mRNAs, tRNAs, lncRNAs, circRNAs, DNA, and mtDNA), proteins, lipids, or signaling molecules [79]. Combining several types of markers in multifactorial assays could enhance the sensitivity of liquid biopsies.

Exosomal CD151 was demonstrated to be significantly upregulated in lung cancer, enabling a distinction between healthy and cancer patients [80]. Li et al. [81] demonstrated the presence of exosomes containing claudin-4 in the peripheral circulation of ovarian cancer patients. In melanoma patients, the level plasma exosomes expressing CD63 and caveolin-1 were shown to be significantly increased [82]. Glypican-1 was enriched in the circulating exosomes of pancreatic ductal adenocarcinoma patients and was shown to determine the tumor size and disease burden [83]. Besides proteins, exosomal miRNAs may also serve as cancer biomarkers. For example, Hannafon et al. [84] revealed that miR-1246 and miR-21 were significantly enriched in the plasma exosomes of breast cancer patients. On the contrary, miR-638 was shown to be significantly decreased in serum exosomes in patients suffering from colon cancer. Moreover, the decrease in miR-638 was more significant in patients at later TNM stages or with liver metastasis, enabling the discrimination more severe cancers from less advanced diseases [85]. GPC1, a cell surface proteoglycan, was significantly enriched in the serum of patients with pancreatic cancer [86]. Tumor-derived exosomes also contain double-stranded DNA (dsDNA), which reflects the entire genome and mutations in tumor cells, as demonstrated by Thakur et al. [87]. This might be particularly relevant in the case of pancreatic cancer, since mutations in *KRAS* and *TP53* can be detected in genomic DNA obtained from serum exosomes derived from pancreatic cancer patients [88,89]. Besides utilizing exosomes as cancer biomarkers, there is also growing interest amongst scientists in engineering exosomes to serve as cell-free therapeutic agents. Important considerations to use exosomes as drug delivery vehicles include the choice of the donor cell type, the type of therapeutic cargo, and the route of administration [90]. Exosome donor cells may derive from model cell lines, MSCs, or other more specialized cell types [91]. Exosomes as drug delivery vehicles possess several advantages over synthetic delivery systems. For instance, they have phospholipid bilayers, allowing them able to fuse directly with the membrane of target cells. Moreover, they can be derived from patients’ cells, reducing the risk of immunological response [92]. Due to their size, they are able to avoid phagocytosis and extravasates through tumor vessels [93]. Exosomes may be engineered to target specific cells, depending on the surface molecules.

Exosomal microRNAs or small interfering RNAs (siRNAs) could be able to target specific mRNAs in recipient cell and affect gene expression [2]. Therefore, these molecules may play a vital role in cancer gene therapy. Shtam et al. [94] utilized exosomes derived from HeLa and HT1080 fibrosarcoma cells, which were loaded with siRNAs targeted against *RAD51* and *RAD52* genes. As a result, targeted genes were silenced in recipient cancer cells, and silencing of *RAD51* caused the reproductive cell death. Similarly, Limoni et al. [95] transduced HEK293T cells with the HER2-specific designed ankyrin repeat protein (DARPin) to target the HER2-overexpressing breast cancer cells (the SKBR3 cell line). Exosomes derived from engineered cells were loaded with siRNA molecules against the *TPD52* gene, an oncogene correlated with SKBR3 cell survival. The treatment of breast cancer cells with engineered exosomes resulted in a 70% downregulation of *TPD52* gene expression [95]. Kamerkar et al. [96] engineered exosomes derived from normal fibroblast-like mesenchymal stem cells to carry siRNAs or shRNAs specific to oncogenic KRAS^G12D^, which is a common mutation in pancreatic cancer. As a result, cancer was suppressed in mouse models and their survival was significantly increased. Moreover, engineered exosomes were more effective than liposomes due to CD47 which facilitated the protection against phagocytosis.

Recently, a new exosome-based strategy to target the anti-apoptotic protein BCL-2 was provided. BCL-2 is frequently present in multiple tumors, mainly in patients with estrogen receptor-positive (ER+) breast cancer. Kaban et al. used BCL-2 siRNA-loaded exosomes derived from genetically modified NK cells to treat ER+ breast cancer, resulting in the increased intrinsic apoptosis pathway in cancer cells, while non-malignant cells were not affected [97]. Similarly, another study has shown that exosomes loaded with PLK-1 siRNAs promoted bladder cell apoptosis by silencing the expression of polo-like kinase 1 (PLK-1), a major driver of cancer cell growth and proliferation [98]. Interestingly, Zhou et al. constructed a dual delivery system based on exosomes derived from bone marrow mesenchymal stem cells (BM-MSC). Exosomes were loaded with galectin-9 siRNAs via electroporation, and their surfaces were modified with the oxaliplatin prodrug as an immunogenic cell death trigger [99]. Treatment with this combined formulation in mice improved pancreatic ductal adenocarcinoma (PDAC) immunotherapy and reversed tumor immunosuppression of M2-like tumor-associated macrophages by disrupting the galectin-9/dectin-1 axis [99].

MicroRNAs are often differentially expressed in cancer cells as compared to healthy tissues, modulating oncogenes and tumor suppressors [100]. Two types of miRNA-based therapies may be distinguished: miRNA replacement and miRNA inhibition [101]. In the first one, exogenous miRNAs are delivered by exosomes to cancer cells, leading to tumor suppression. The second strategy aims to inhibit tumor-promoting miRNAs via specific miRNA inhibitors or synthetic anti-miRNA oligonucleotides (AMOs) [101]. To date, a great amount of research has been conducted.

MiR-146b-5p was previously demonstrated to suppress the expression of the EGFR in human glioblastoma cell lines and to reduce the in vitro migration and invasion of glioma by Katakowski et al. [102] Therefore, in a subsequent study, they transfected BM-MSCs with a miR-146b expression plasmid and collected the released exosomes. Subsequently, exosomes containing miR-146b were injected intratumorally into the rat model of a primary brain tumor, which significantly reduced glioma xenograft growth [103]. A similar approach was undertaken by Munoz et al. [104] who transfected MSCs with anti-miR-9 and collected the released exosomes. Importantly, miR-9 was shown to influence the expression of the multidrug transporter, P-glycoprotein, which led to drug resistance. Next, glioblastoma multiforme cells were treated with exosomes, which resulted in the reversed expression of multidrug transporters and in sensitization to temozolomide, leading to cell death. In terms of prostate cancer, Kosaka et al. [105] conducted a study utilizing exosomes from the non-cancerous prostate cell-line PNT-2 and a hormone-insensitive prostatic carcinoma cell line PC-3M-luc. The treatment with healthy cell-derived exosomes resulted in the inhibition of cancerous cell growth and proliferation. Such an effect was ascribed to miR-143, a tumor-suppressive miRNA released by PNT-2 cells. Specific miRNAs could also be delivered to breast cancer cells, as indicated by Ohno et al. [106]. To target the epidermal growth factor (EGFR)-expressing breast cancer cells, donor HEK293 cells were engineered to express GE11, which binds specifically to the EGFR. Then, the exosomes were collected from the culture medium and transfected with let-7a, since this miRNA was implicated in tumor suppression. Transfected exosomes were then intravenously injected to RAG2−/− mice with EGFR-expressing xenograft cancer tissue. This resulted in tumor suppression, suggesting that specifically modified exosomes may be a promising vehicle for drug delivery [106].

Besides siRNAs and microRNAs, various chemotherapeutics can be loaded into exosomes, which is particularly relevant in the case of cancer. Exosomal delivery may reduce the overall toxicity of chemotherapy because the exosomes are engineered to specifically target tumor cells. Tian et al. [93] utilized immature dendritic mouse cells, which were engineered to express lysosome-associated membrane glycoprotein 2b (Lamp2b) fused with targeting peptide for αv integrins. The exosomes released by these cells were then collected and loaded with doxorubicin via electroporation. The treatment of αv integrin-positive MDA-MB-231 cells with doxorubicin-loaded exosomes resulted in inhibited cell proliferation with an efficiency comparable to free doxorubicin. In vivo studies revealed that the intravenous injection of doxorubicin-loaded exosomes lead to the targeted delivery of doxorubicin into tumor tissues and the inhibition of tumor growth without overt toxicity [93]. Bellavia et al. [107] engineered HEK293T cells to express exosomal protein LAMP2B fused with a fragment of interleukin 3 (IL3), since the receptor of IL3 is overexpressed in chronic myeloid leukemia. Such modified exosomes were loaded with imatinib or siRNAs against the *BCR-ABL* gene, which is associated with acquired drug resistance. The authors revealed that exosomes were able to specifically target chronic myeloid leukemia cells. Furthermore, the exosomes inhibited cancer cell growth both in vitro and in vivo [107]. An interesting approach was undertaken by Liang et al. [108], who engineered exosomes for the targeted co-delivery of miRNA inhibitors and chemotherapeutics. Patients with colorectal cancer often exhibit resistance to 5-fluorouracil (5-FU), due to the regulatory functions of miR-21. Therefore, 5-FU-resistant HCT-116 cells were loaded with the miR-21 inhibitor and 5-FU, which led to the significant downregulation of miR-21 expression and cell cycle arrest. Moreover, the drug resistance was reversed and cytotoxicity was enhanced, as compared with the treatment using the miR-21 inhibitor or 5-FU alone [108].

Similarly, Zhan et al. modified blood exosomes by co-loading hydrophobic doxorubicin and the cholesterol-modified miRNA-21 inhibitor (miR-21i) into the lipid bilayer and binding the magnetic-targeting molecules and L17E peptides to the membrane of exosomes. This multifunctional blood exosome-based delivery system was found to significantly inhibit cancer cell proliferation in mice bearing the U87 tumor. Magnetic molecules and L17E peptides, used in modified exosomes, remarkably improved the delivery efficiency of encapsulated cargo to cancer cells [109].

Although the possibility to utilize exosomes as cancer biomarkers or cell-free therapeutic agents seems promising, it is important to consider their heterogeneity and, therefore, develop a technology for their appropriate characterization. The choice of source of exosomes suitable for drug delivery vehicles still remains to be investigated. Further in vivo studies in the clinical environment are required to establish an accurate concentration of exosomes exerting therapeutic effects in cancer, as well as to develop efficient methods of drug loading and targeted engineering.

## 5. The Role of Exosomes in Drug Resistance in Oncology

Since exosomes are known to serve as mediators of cell-to-cell communication, it has been suggested that they might be involved in cancer drug resistance. Exosome-mediated information transfer may occur through a transfer of drug transporter proteins (MRP1 or P-glycoprotein [110]) or a transfer of miRNAs. An example of such action was provided by Chen et al. [111] in breast cancer cell lines. The authors revealed that exosomes derived from variants that were resistant to cancer cell lines (MCF-7/Adr and MCF-7/Doc) were able to significantly modulate cell cycle and drug-induced apoptosis due to miRNA action. miR-222 was especially associated with the alteration of gene expression in sensitive MCF-7/S cells. Therefore, the authors demonstrated that drug-resistant breast cancer cells may spread this resistance capacity to sensitive cells via exosomes and exosomal miRNAs [111]. Hu et al. [112] revealed that CAF-derived exosomes contribute to chemoresistance. Exosomes isolated from the conditioned medium of fibroblasts were shown to promote the percentage, clonogenicity, and tumor growth of cancer stem cells (CSCs) during treatment with oxaliplatin or 5-fluoroacil. Exosomal-mediated drug resistance was decreased via the inhibition of fibroblast exosome secretion. A study conducted by Deng et al. [113] revealed that colorectal-cancer-associated lncRNA (CCAL) might be involved in the acquisition of resistance to oxaliplatin by colorectal cancer cells. Functional studies revealed that CCAL was transferred to colorectal cancer cells by exosomes derived from CAFs. CCAL may have led to apoptosis suppression, chemoresistance, and β-catenin pathway activation both in vivo and in vitro [113]. The resistance of ovarian cancer cells to platinum-based therapy was associated with the increased expression of annexin 3 released in exosomes, as indicated by Yin et al. [114]. The elevated expression of plasma gelsolin was associated with chemoresistance and poorer overall survival of patients with ovarian cancer. Plasma gelsolin was shown to be transferred via exosomes to chemosensitive ovarian cancer cells conferring cisplatin resistance [115]. The transfer of miR-365 in exosomes derived from tumor-associated macrophages (TAMs) was demonstrated to regulate resistance to gemcitabine in pancreatic ductal adenocarcinoma cells (PDACs). MiR-365 was shown to induce the enzyme-inactivating gemcitabine, known as the cytidine deaminase; therefore, TAM-derived exosomes significantly decreased the sensitivity of PDAC cells to gemcitabine [116].

Exosomes may facilitate the removal of chemotherapeutics from cancer cells and their microenvironment via the direct or indirect regulation of drug efflux pumps. Muralidharan-Chari et al. [117] described microvesicles involved in gemcitabine resistance in pancreatic cancer cells. Gemcitabine was shown to trigger the release of microvesicles and the inhibition of this release resulted in the sensitization of pancreatic cancer cells to gemcitabine both in vitro and in vivo. Therefore, gemcitabine was removed from the cells via microvesicles. However, in case of microvesicles released by a drug-resistant pancreatic cancer cell line, gemcitabine remains trapped in microvesicles, while microvesicles released from the drug-sensitive cancer cell line remove gemcitabine back to the microenvironment due to the increased expression of efflux proteins (MRP-5 and P-gp). These results highlight the importance of microvesicle release in acquiring drug resistance [117]. Ning et al. [118] revealed that exosomes secreted by adriamycin-resistant human breast cancer cells (MCF7/ADM) were able to transfer ubiquitin carboxyl terminal hydrolase-L1 (UCH-L1) and P-gp into the extracellular matrix. Subsequently, these proteins were able to integrate into adriamycin-sensitive human breast cancer cells (MCF7/WT), transferring the chemoresistance phenotype [118].

The involvement of exosomes in cancer cell chemoresistance may include the induction of the epithelial-to-mesenchymal transition (EMT), which is associated with the upregulation of anti-apoptotic pathways and drug efflux pumps [119]. MiR-155 was demonstrated to be particularly relevant in the EMT and breast cancer therapy resistance. Santos et al. [120] revealed that exosomes secreted by CSCs and resistant breast cancer cells contained miR-155, which was transferred to the recipient sensitive cells. It appears that the acquisition of resistance phenotype occurs via increasing markers of the EMT, such as B lymphoma Mo-MLV insertion region 1 homolog (BMI1), SLUG, SNAIL, or SOX9. Similarly, in the case of epithelial ovarian cancer cells, exosomes were shown to participate in the transfer of chemoresistance phenotypes from the platinum-resistant OVCAR10 cell line to the platinum-sensitive A2780 cell line. Chemoresistance occurred due to increased EMT and somatic mutations in *SMAD4* (*Mothers against decapentaplegic homolog 4*) [121]. Similar results were obtained in gastric cancer cell lines, since the paclitaxel-resistant MGC-803R cell line was shown to secrete exosomes enriched in miR-155-5p, which were taken up by paclitaxel-sensitive MGC-803S cells. This led to EMT induction and the downregulation of GATA binding protein 3 (GATA3) and tumor protein p53-inducible nuclear protein 1 (TP53INP1) [122].

Exosomes also participate in drug resistance via alterations in signal transduction. Hu et al. [123] demonstrated that fibroblast-derived exosomes promote chemoresistance in colorectal cancer cells (CRCs) due to the dedifferentiation of CRCs to cancer stem cells (CSCs), which are chemotherapy-resistant. Specifically, exosomal Wnts were shown to increase Wnt activity and drug resistance in CRC, leading to CRC dedifferentiation.

**Figure 4:**
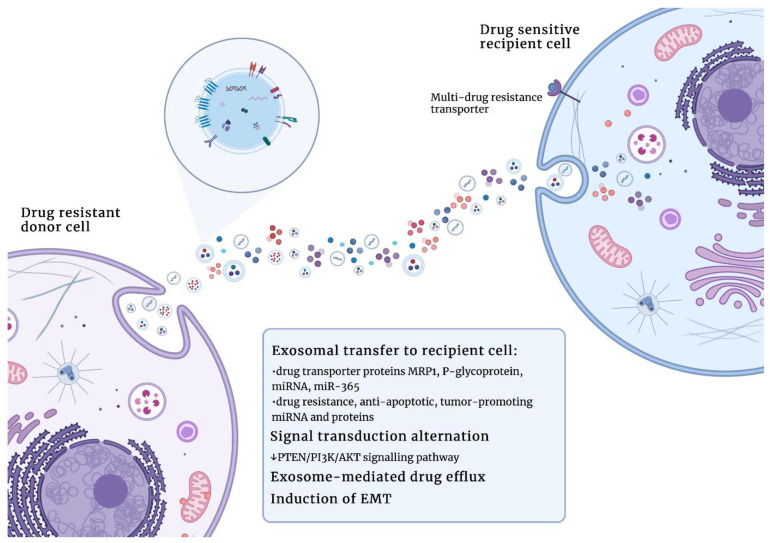
Exosomes play an important role in the acquisition of drug resistance in cancer. Abbreviations: MRP1 (multidrug resistance protein 1); PTEN (phosphatase and tensin homolog); PI3K (phosphoinositide 3-kinase); AKT (protein kinase B); EMT (epithelial–mesenchymal transition). Figures were created with BioRender.com (accessed on 1 November 2022).

Besides facilitating resistance to typical chemotherapeutic drugs, exosomes were also demonstrated to participate in cancer cell resistance to hormonal therapy. Semina et al. [124] demonstrated that exosomes collected from the estrogen-dependent MCF-7 breast cancer cell subline that is resistant to anti-estrogen drugs (such as tamoxifen or metformin) caused partial resistance in parent MCF-7 cells after 14 days of treatment. Resistance was probably caused by a decrease in estrogen receptor α (Erα) activity, and the activation of anti-apoptotic and EMT-associated transcription factors, such as AP-1, NF-κB, and SNAIL1. Importantly, the cell sensitivity to drugs was not restored after culture in exosome-free media, suggesting that acquired resistance is irreversible [124]. The role of exosomes in the acquisition of drug resistance in cancer is schematically presented in Figure 4.

## 6. The Clinical Application of Exosomes in Humans

Given that exosomes play many important and complex functions in the human body, they can be utilized in multiple ways in the clinical environment. The clinical application of exosomes was investigated using the website clinicaltrials.gov, searching the terms “exosome” and “exosomes” (278 results) and “exosomes cancer” (119 results), which concern 176 conditions, such as prostatic, colorectal, or lung neoplasms. The amount of obtained results clearly indicates that this topic is of interest amongst scientists. Exosomes are particularly promising when utilized as drug delivery vehicles that could be fabricated for a specific patient and specific disease, evading any potential immune response. However, in terms of clinical trials utilizing exosomes in cancer, most of them concern the use of exosomes as diagnostic biomarkers. The findings of these trials are listed in Table 1.

Exosomal CD133 was suggested to be a potential prognostic biomarker in advanced pancreatic cancer patients by Sakaue et al. [125]. Exosomes derived from the malignant ascites of nineteen pancreatic cancer patients exhibited an increased expression of CD133, as compared to exosomes derived from patients with gastric cancer or liver cirrhosis. Moreover, an equilateral correlation between the presence of highly glycosylated CD133 and overall survival has been indicated by Western blot analysis. Therefore, it seems that highly glycosylated CD133 in ascite-derived exosomes could serve as a biomarker for better prognoses in pancreatic cancer patients [125]. Pan et al. [126] attempted to distinguish specific exosomal miRNAs as tumor markers in epithelial ovarian cancer. Exosomes were collected from the blood plasma of 106 epithelial ovarian cancer patients and 8 ovarian cystadenoma patients, and their miRNA content was compared with miRNAs contained in exosomes collected from healthy women. MiR-21, miR-100, miR-200b, and miR-320 were shown to be upregulated in exosomes derived from epithelial ovarian cancer patients. The level of miR-200b especially increased and was correlated with the tumor marker CA125. In the case of ovarian cystadenoma, the levels of exosomal miR-23a and miR-92a were downregulated [126].

Huang et al. [127] undertook a different approach and aimed to focus on urinary exosomes as a biomarker in thyroid cancer. They collected urine from sixteen patients with papillary thyroid carcinoma and follicular thyroid carcinoma and analyzed its exosomal protein content, especially focusing on thyroglobulin and galectin-3. Samples from each patient were collected four times: before thyroidectomy, immediately after operation, and three and six months after operation. Urinary exosomal thyroglobulin showed an increasing trend in post-operative patients as compared to serum thyroglobulin, suggesting the probable recurrence of cancer. Importantly, in some patients, serum thyroglobulin was not detectable after the surgery, whereas urinary exosomal thyroglobulin was present, suggesting that it may serve as a potential biomarker of thyroid cancer recurrence [127].

Studies concerning the utilization of exosomes as therapeutic agents and delivery carriers are performed mostly in animals; however, several studies have been conducted in humans. Unfortunately, to date, there are not many published results of these trials, especially not the recent ones. The findings of such trials are listed in Table 2.

Escudier et al. [128] conducted a phase I clinical trial utilizing autologous dendritic-cell-derived exosomes in metastatic melanoma patients. Fifteen stage III/IV melanoma patients received four intradermal and subcutaneous exosome vaccinations at 1 week intervals. Exosomes were purified from dendritic cell culture supernatants and loaded with MHC class I or II peptides. As a result of exosomal treatment, no major toxicity was observed; however, slight inflammatory reactions occurred at vaccines sites. One patient exhibited a partial response according to the RECIST criteria. However, the main outcome of this study was that the exosomal vaccine was safe for patients with metastatic melanoma [128].

Similarly, Morse et al. [129] loaded dendritic-cell-derived exosomes with the MAGE tumor antigens and used such a vaccine in the case of advanced non-small cell lung cancer (NSCLC). Thirteen patients with stage III/IV NSCLC received four doses of the vaccine at weekly intervals and nine completed the therapy. No serious adverse events were observed; therefore, such therapy was well tolerated in NSCLC patients. Three out of nine patients developed specific immune responses and the lytic activity of NK cells was upregulated [129]. Subsequently, a phase II clinical trial in unresectable NSCLC patients was conducted with the use of IFNγ maturated dendritic-cell-derived exosomes. These exosomes were loaded with MHC class-I- and class-II-restricted cancer antigens and injected to twenty-two patients. The median overall survival was 15 months. There was no objective tumor response according to RECIST criteria. However, an increase in NKp30-dependent NK cell functions was observed [130].

Dai et al. [131] conducted a phase I clinical trial utilizing the ascite-derived exosomes combined with the granulocyte–macrophage colony-stimulating factor (GM-CSF) in colorectal cancer patients. Forty patients received four subcutaneous injections of exosomes alone or exosomes combined with GM-CSF at weekly intervals. Both therapies were safe and no serious adverse events were observed. Moreover, the treatment with exosomes combined with GM-CSF was able to induce a tumor-specific antitumor cytotoxic T cell response [131].

Although there are many clinical trials registered utilizing exosomes as biomarkers or therapeutic agents, only a few have published the results. It seems that the enhancement of diagnosis in cancer would be a primary goal for exosome utilization for now, since their engineering to serve as therapeutic agents and carriers is much more complex and is still in its infancy. It is interesting to notice that many of the clinical studies concerning treating cancer with exosomes utilize exosomes derived from dendritic cells, which exert immunomodulatory functions and may be used to target a specific type of cancer. All interventions listed in Table 2 were demonstrated to be safe for the patients, which raises the possibility of further developing this type of research. These interventions were more focused on enhancing the immune response rather than specifically delivering a drug; however, aforementioned animal and in vitro studies clearly show that this might be a possibility in the future. Studies listed in Table 1 indicate that exosomes derived from various sources, such as urine or blood, could be helpful in predicting cancer’s recurrence or in diagnosing the type of cancer in humans.

## 7. Conclusions

Exosomes play a major function in cell-to-cell communication, both in pathological and physiological conditions. Exosomes may facilitate tumor progression, angiogenesis, metastasis, and drug resistance. Therefore, their utilization in cancer therapies seems to be particularly interesting, considering exosomal physical properties and their accessibility. Exosomes may serve as a disease biomarker, allowing cancer progression or cancer treatment to be monitored,. Constantly developing methods of exosome isolation, purification, and modification create an opportunity to further optimize their utilization in clinical environment. Indeed, numerous clinical trials employing exosomes in cancer treatment as drug delivery vehicles or as potential novel biomarkers arise, indicating that this topic is of interest amongst scientists. However, it is important to establish highly a standardized method of exosomal utilization, in accordance with GMP and GCP. It seems that utilizing exosomes in cancer diagnosis would be the primary goal for now, since engineered exosomes used for drug delivery or immunotherapy would be associated with more interference with the human organism. Preclinical studies provide promising results; however, further studies are required to evaluate the influence of such exosomes on human body and cancer treatment.

Take-home messages are as follows:

- Exosomes are nanoscale biovesicles produced by most mammalian cells that play a pivotal role in cell–cell communication in both physiological and pathological conditions;
- Exosomes can modulate the tumor microenvironment, leading to tumor progression, angiogenesis, metastasis, and drug resistance by transferring information to sensitive cells;
- Taking into account the properties and accessibility of exosomes, they can serve as biomarkers for cancer detection and progression, as well as drug delivery vehicles, specifically targeting cancer cells.

## Figures and Tables

**Table 1:** Clinical studies utilizing exosomal content as biomarkers of various types of cancer.

Cancer Type	Exosomal Biomarker	Specimen Origin	Function of Biomarker	Number of Patients	Reference
Pancreatic cancer	CD133	Malignant ascites	Upregulated in pancreatic cancer patients; discrimination from gastic cancer or liver cirrhosis patients; highly glycosylated CD133 for better prognoses in pancreatic cancer	19	Sakaue et al., 2019 [125]
Epithelial ovarian cancer	miR-21, miR-100, miR-200b, miR-320	Blood plasma	Upregulated in epithelial ovarian cancer patients; discrimination from healthy women	106	Pan et al., 2018 [126]
Ovarian cystadenoma	miR-23a, miR-92a	Blood plasma	Downregulated in ovarian cystadenoma patients; discrimination from healthy women	8	Pan et al., 2018 [126]
Thyroid cancer	Thyroglobulin	Urine	Increased cases of possible cancer recurrence	16 papillary thyroid carcinoma and follicular thyroid carcinoma patients	Huang et al., 2020 [127]

**Table 2:** Clinical studies utilizing exosomes as therapeutic agents in cancer.

Cancer Type	Source of Exosomes	Modification of Exosomes	Effects of the Therapy	Number of Patients	Reference
Melanoma	Dendrtic cells	Exosomes loaded with MHC class I or II peptides	No major toxicity was observed after intradermal and subcutaneous injections. A partial response in one patient (according to the RECIST criteria).	15	Escudier et al., 2005 [128]
Non-small cell lung cancer	Dendritic cells	Exosomes loaded with MAGE tumor antigens	No serious adverse events were observed.	13	Morse et al., 2005 [129]
	Dendritic cells	IFNγ-maturated exosomes loaded with MHC class I and II antigens	No objective tumor response (according to the RECIST criteria). An increase in NKp30-dependent NK cell functions.	22	Besse et al., 2016 [130]
Colorectal cancer	Ascites	Exosomes alone or combined with GM-CSF	No serious adverse events were observed. Exosomes combined with GM-CSF induced antitumor cytotoxic T cell response.	40	Dai et al., 2008 [131]

## Data Availability

Data sharing not applicable.
